# Transcriptome dataset of sago palm in peat soil

**DOI:** 10.1016/j.dib.2022.107908

**Published:** 2022-02-03

**Authors:** Wei-Jie Yan, Hasnain Hussain, Hung Hui Chung, Norzainizul Julaihi, Rina Tommy

**Affiliations:** a Centre for Sago Research (CoSAR), Faculty of Resource Science and Technology, Universiti Malaysia Sarawak, 94300 Kota Samarahan, Sarawak, Malaysia; b Faculty of Resource Science and Technology, Universiti Malaysia Sarawak, 94300 Kota Samarahan, Sarawak, Malaysia; c Land Custody and Development Authority, Level 4, 8 & 12, Wisma Satok, Jalan Satok, 93400 Kuching, Sarawak, Malaysia

**Keywords:** Sago palm, *Metroxylon sagu*, RNA sequencing, Transcriptome, Non-trunking

## Abstract

Sago palm (*Metroxylon sagu* Rottb.) is an important agricultural starch-producing palm that contributes to Malaysia's economics, especially in the State of Sarawak, Malaysian Borneo. In this palm tree, the central part of the plant storage-starch. Under normal condition, sago palm develop its trunk after 4-5 years being planted. However, sago palms planted on deep-peat soil failed to develop their trunk even after 17 years of being planted. This phenomenon is known as ‘non-trunking’, which eliminates the economic value of the palms. Numerous research has been done to address the phenomenon, but the molecular mechanisms of sago palm responding toward the responsible stresses are still lacking. Therefore, in this study, leaf samples were collected from trunking (normal) and non-trunking sago palms planted on peat soil for total RNA extraction, followed by next-generation sequencing using the BGISEQ-500 platform. The raw reads were cleaned, and *de novo* assembled using TRINITY software package. A total of 40.11 Gb bases were sequenced from the sago palm leaf samples. The assembled sequence produced 102,447 unigenes, with N50 score 1809 bp and GC ratio of 44.34%. The alignment of unigenes with seven functional databases (NR, NT, GO, KOG, KEGG, SwissProt and InterPro) resulted in the annotation of 65,523 (63.96%) unigenes. Functional annotation results in the detection of 46,335 coding DNA sequences by Transdecoder. A total of 30,039 simple-sequence repeats distributed on 21,676 unigenes were detected using Primer3 software, and 2355 transcription factor coding unigenes were predicted using getorf and hmmseach software. This work is registered under NCBI BioProject PRJNA781491. The raw RNA sequencing data are available in Sequence Read Archive (SRA) database with accession numbers SRX13165895, SRX13165896, SRX13165897, SRX13165898, SRX13165899, and SRX13165900. Gene expression and annotation information are accessible in public functional genomics data repository Gene Expression Omnibus (GEO) with accession number GSE189085.

## Specifications Table

**Table:** 

Subject	Biological sciences; Omics: Transcriptomics
Specific subject area	Trunk development of sago palm under stress
Type of data	Transcriptomics data (raw RNA sequence reads, gene expression and sequence annotation)
How the data were acquired	BGISEQ-500 platform
Data format	Raw: *fastq.gz files Assembly: *Unigene.fa.gz files Processed Data: *gene.fpkm.txt.gz files
Description of data collection	Total RNA was extracted from trunking and non-trunking sago palm (*M. sagu*) leaf tissue, mRNA library preparation and then was sequenced using the BGISEQ-500 platform
Data source location	Dalat Sago Plantation, Mukah, Sarawak, Malaysia GPS location are listed in Table 1)
Data accessibility	Repository name: NCBI's Gene expression omnibus (GEO) Data identification number: GSE189085 Direct URL to data: https://www.ncbi.nlm.nih.gov/geo/query/acc.cgi?acc=GSE189085 Repository name: NCBI's Sequence Read Archive (SRA) Sample ID: GSM5694359 (ST1: Trunking Sample 1) Data identification number: SRX13165895 Direct URL to data: https://www.ncbi.nlm.nih.gov/sra/SRX13165895[accn] Repository name: NCBI's Sequence Read Archive (SRA) Sample ID: GSM5694360 (ST4: Trunking Sample 4) Data identification number: SRX13165896 Direct URL to data: https://www.ncbi.nlm.nih.gov/sra/SRX13165896[accn] Repository name: NCBI's Sequence Read Archive (SRA) Sample ID: GSM5694361 (ST5: Trunking Sample 5) Data identification number: SRX13165897 Direct URL to data: https://www.ncbi.nlm.nih.gov/sra/SRX13165897[accn] Repository name: NCBI's Sequence Read Archive (SRA) Sample ID: GSM5694362 (NT7: Trunking Sample 7) Data identification number: SRX13165898 Direct URL to data: https://www.ncbi.nlm.nih.gov/sra/SRX13165898[accn] Repository name: NCBI's Sequence Read Archive (SRA) Sample ID: GSM5694363 (NT8: Trunking Sample 8) Data identification number: SRX13165899
	Direct URL to data: https://www.ncbi.nlm.nih.gov/sra/SRX13165899[accn] Repository name: NCBI's Sequence Read Archive (SRA) Sample ID: GSM5694364 (NT9: Trunking Sample 9) Data identification number: SRX13165900 Direct URL to data: https://www.ncbi.nlm.nih.gov/sra/SRX13165900[accn]

## Value of the Data

• This data is useful for the scientific community as it provides insights into the transcriptome of *M. sagu*.
• This data provides a comprehensive transcriptomic expression using pair-end sequencing with two sets of samples with three biological replicate datasets, each to comprehend gene expression contributing to the non-trunking phenomenon in *M. sagu*.
• Researchers involved with the work related to the omics study of *M. sagu* could also benefit from this data as cross-references information to support their findings.

## 1 Data Description

Sago palm grows through a series of developmental stages, which takes up to twelve years to be ready for the harvest. *M. sagu* generates suckers (soboliferous) every 18 months as the successor of the mother plant, which dies after fruiting (hapaxanth). Mature sago palm yields 15–25 metric tons of air-dried starch per hectare at the end of an 8-year growth cycle under good condition [1]. The advantages of sago palm as a starch-producing crop that grows in peat soil with seasonal waterlogged has triggered the Land Custody and Development Authority Sarawak [2] to initiate the commercial plantation in Mukah, Sarawak in 1987. However, there was the occurrence of non-trunking sago palms even after ten years of cultivation. The non-trunking sago palm reduced starch yield per hectare of land, resulting in the instability of the sago starch market. It reduced the plantation income, consequently restricting the development of sago industries and loss of confidence in this palm by the potential or current sago palm farmers [3].

Numerous studies were performed to address the non-trunking sago palm problem such as soil physicochemical properties [2], soil microbiome [4] and molecular studies [5], [6], [7]. The general outcome of the studies revealed that the mineral deficiency causes the non-trunking in sago palm, but how this deficiency affects sago development remains unanswered. Currently, several research studies of this palm in genomics and proteomics are being conducted. In conjunction with those studies, this study utilises transcriptome analysis to compare the gene expression between the trunking and non-trunking sago palm leaf tissue to highlight the differential expressed genes and their correlation with the non-trunking phenomenon in sago palm.

The information in this article includes the transcriptomics of trunking sago palm (control) and non-trunking sago palm (target of interest) from peat soil. The global gene expression between the trunking and non-trunking sago palm was evaluated by differential expressed genes analysis. The files of the transcriptome dataset, which generated from 6 libraries of raw data and 2 sets of processed data, were submitted to Sequence Read Archive (SRA) and Gene Expression Omnibus (GEO) NCBI database.

## 2 Experimental Design, Materials and Methods

### 2.1 Sample collection

Sago palm leaf tissues were used in this study. They were six samples consisting of three biological replicates of 2 phenotypes. All the samples were collected from Dalat Sago Plantation in Mukah, Sarawak (Table 1). The samples were wiped with a kitchen towel containing 70% ethanol to remove debris. The samples were then stored in containers followed by snap-freeze in liquid nitrogen. The samples were kept in liquid nitrogen before being transferred into a ‒80 °C freezer for long-term storage.

**Table 1:** Samples Global positioning system (GPS).

Morphology	Sample	GPS (WGS84 datum)
Non-Trunking (Target of interest)	SN7	+2° 49′ 40.5″, +111° 50′ 25.8″
	SN8	+2° 49′ 40.7″, +111° 50′ 25.6″
	SN9	+2° 49′ 40.4″, +111° 50′ 26.3″
Trunking (Control)	ST1	+2° 51′ 07.7″, +111° 49′ 35.9″
	ST4	+2° 51′ 08.0″, +111° 49′ 35.5″
	ST5	+2° 51′ 07.8″, +111° 49′ 36.4″

### 2.2 RNA extraction and RNA-seq information

Total RNA of the six samples were extracted using CTAB protocol and sequenced using BGISEQ-500 platform. Trunking and non-trunking sago palm (*M. sagu*) transcriptome were successfully sequenced, and the raw RNA sequence reads were deposited in NCBI's Sequence Read Archive (SRA) database with the accession numbers SRX13165895, SRX13165896, SRX13165897, SRX13165898, SRX13165899, and SRX13165900.

The total RNA samples were subjected to mRNA enrichment before the RNA sequencing. About 40.11 Gb bases raw sequence reads of the six RNA samples were successfully generated using the BGISEQ-500 sequencing platform. The raw reads containing more than 5% unknown *N* base, adaptor-polluted and more than 20% of bases in the total read with a quality score lower than 15 were then cut-off, and the remaining reads are characterised as clean reads. The clean read ratio exceeded 95% with high accuracy reflected by *Q* score Q30 (equivalent to the probability of an incorrect base call of 1 in 1000 times) above 90% of the reads and Q20 (equivalent to the probability of an incorrect base call of 1 in 100 times) above 95% of the reads (Refer Table 2).

**Table 2:** Clean reads quality metrics.

Sample	Total Raw Reads (Mb)	Total Clean Reads (Mb)	Total Clean Bases (Gb)	Clean Reads Q20 (%)	Clean Reads Q30 (%)	Clean Reads Ratio (%)
ST1	69.96	66.82	6.68	97.88	91.45	95.51
ST4	69.96	66.97	6.7	98.09	92.07	95.72
ST5	69.96	66.68	6.67	97.76	91.14	95.31
SN7	69.96	66.66	6.67	98	91.78	95.27
SN8	69.41	66.62	6.66	97.94	91.59	95.99
SN9	69.96	67.33	6.73	97.98	91.63	96.24

Keys; Sample: Sample name

Total Raw Reads(Mb): The reads amount before filtering

Total Clean Reads(Mb): The reads amount after filtering

Total Clean Bases(Gb): The total base amount after filtering

Clean Reads Q20(%): The rate of bases which quality is greater than 20 value in clean reads

Clean Reads Q30(%): The rate of bases which quality is greater than 30 value in clean reads

Clean Reads Ratio(%): The ratio of the amount of clean reads

### 2.3 *De novo* assembly

**Fig. 1:**
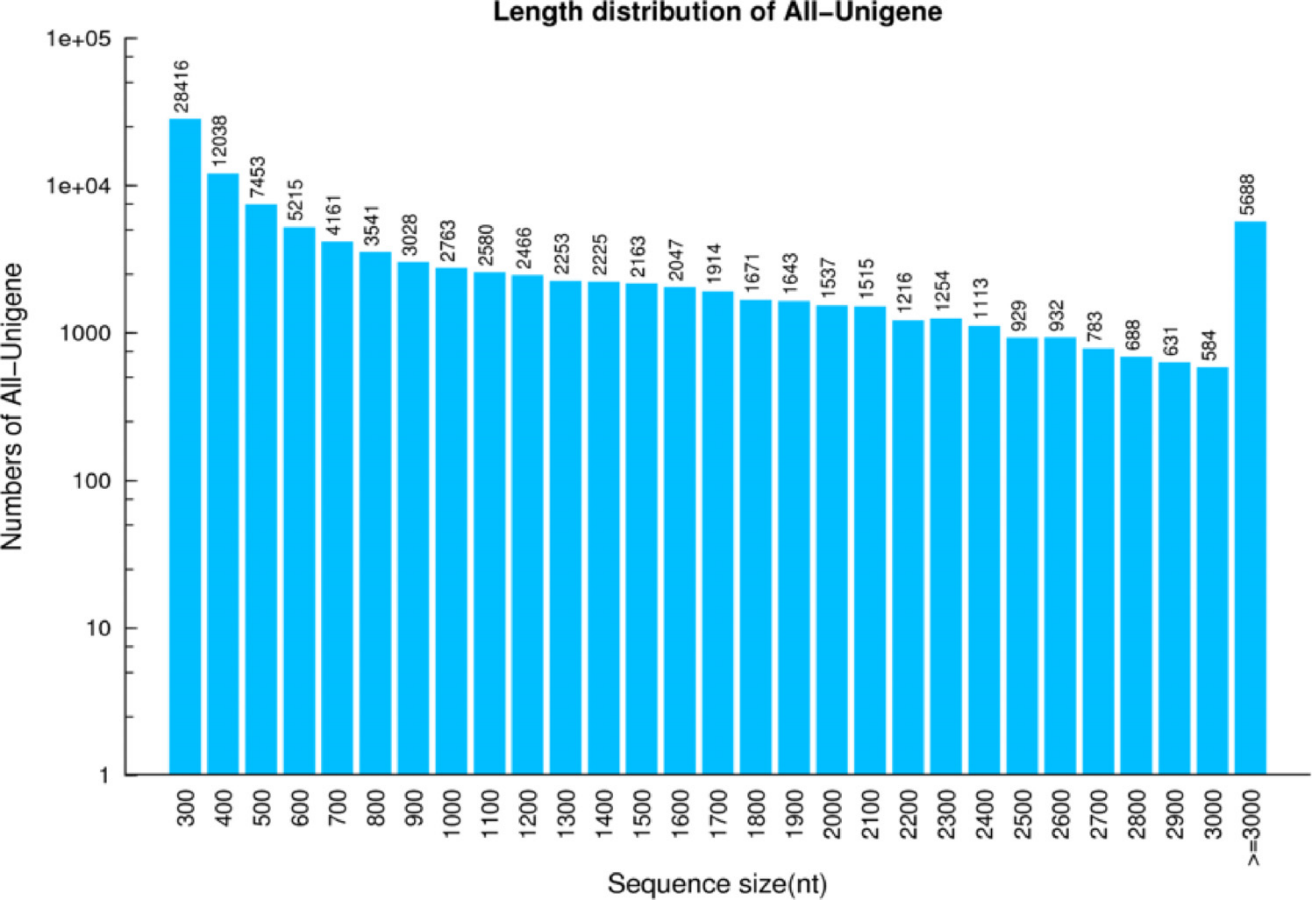
Unigene length distribution. X axis represents the length of Unigenes. Y axis represents the number of unigenes.

The clean reads were then *de novo* assembled using trinity software and generated the reference sequence (Table 3). Reference sequences were then undergone abundance screening using TIGR gene indices clustering tools (TGICL) to obtain unique gene (Unigene) sequences (Table 4; Fig. 1).

**Table 3:** Quality metrics of transcripts.

Sample	Total Number	Total Length	Mean Length	N50	N70	N90	GC(%)
ST1	75730	56765699	749	1334	740	283	45.54
ST4	94989	73822623	777	1447	778	285	44.11
ST5	84578	65560398	775	1430	776	285	44.67
SN7	68068	55890222	821	1472	860	309	45.44
SN8	91100	65484623	718	1303	684	270	44.75
SN9	97205	78047560	802	1503	821	293	43.65

Keys; Sample: Sample name

Total Number: The total number of transcripts

Total Length: The read length of transcripts

Mean Length: The average length of transcripts

N50: The N50 length is used to determine the assembly continuity, the higher the better. N50 is a weighted median statistic that 50% of the total length is contained in Unigenes that are equal to or larger than this value;

N70: Similar to N50

N90: Similar to N50

GC(%): the percentage of G and C bases in all transcripts

**Table 4:** Quality metrics of unigenes.

Sample	Total Number	Total Length	Mean Length	N50	N70	N90	GC(%)
ST1	53270	45270441	849	1409	847	332	45.64
ST4	68390	59838729	874	1522	889	333	44.17
ST5	60537	52810113	872	1506	881	333	44.72
SN7	48542	44955041	926	1542	973	367	45.51
SN8	64137	52366130	816	1390	800	316	44.82
SN9	70377	63341038	900	1570	924	342	43.69
All-Unigene	102447	103410779	1009	1809	1122	382	44.34

Keys; Sample: Sample name

Total Number: The total number of transcripts

Total Length: The read length of transcripts

Mean Length: The average length of transcripts

N50: The N50 length is used to determine the assembly continuity, the higher the better. N50 is a weighted median statistic that 50% of the total length is contained in Unigenes that are equal to or larger than this value;

N70: Similar to N50

N90: Similar to N50

GC(%): the percentage of G and C bases in all transcripts

### 2.4 Unigene functional annotation

**Fig. 2:**
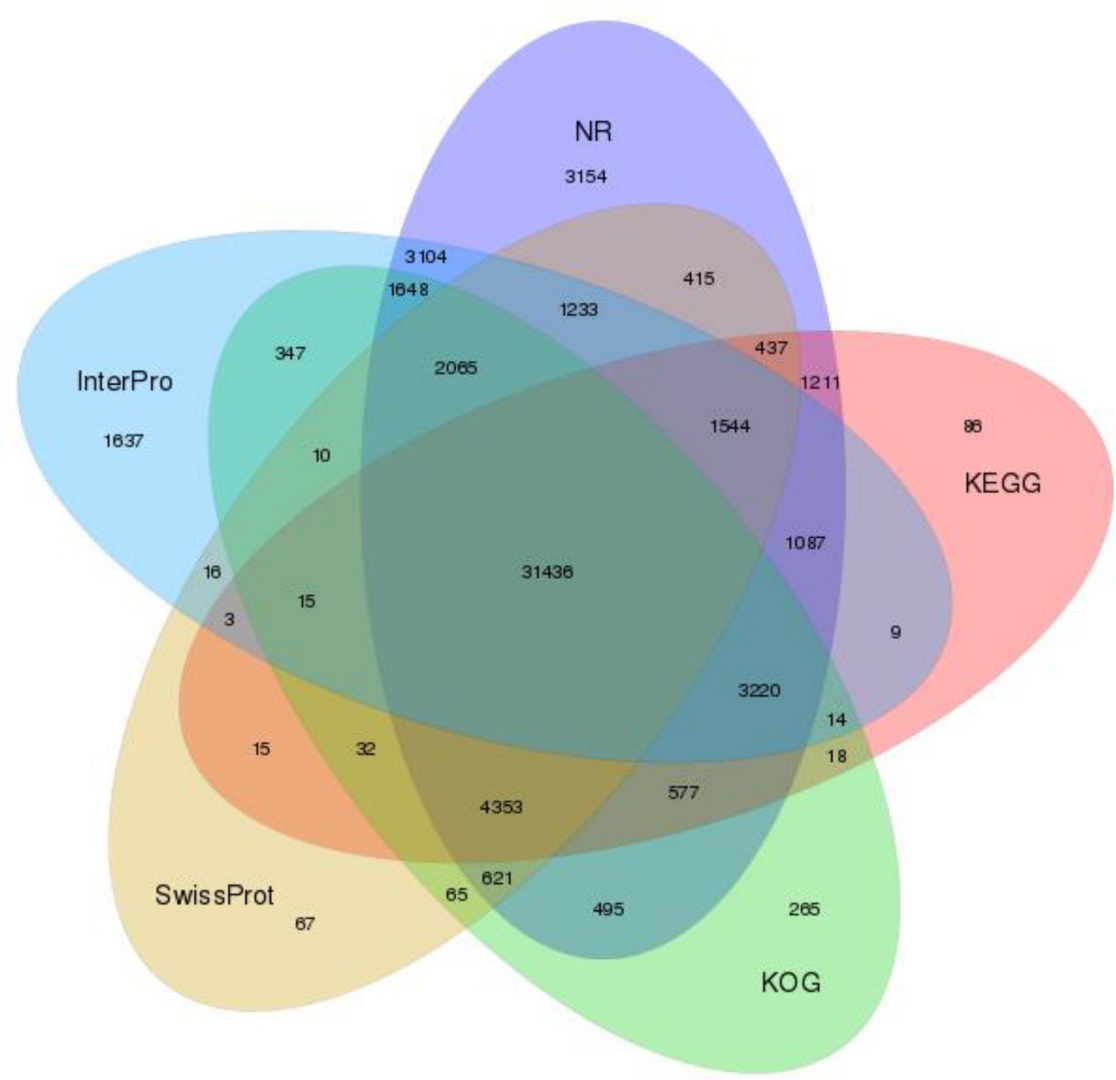
Venn diagram between NR, KOG, KEGG, Swissprot and Interpro.

After assembly, the Unigenes were functionally annotated with seven functional databases, namely; NCBI protein database (NR), NCBI nucleotide database (NT), Gene Ontology (GO), Eukaryotic Orthologous Groups of proteins (KOG), Kyoto Encyclopedia of Genes and Genomes (KEGG), Swiss-Prot, a curated protein sequence database of UniProt, and InterPro (Table 5; Fig. 2). Unigene annotation and expression information are deposited in NCBI's Gene Expression Omnibus (GEO) with accession number GSE189085.

**Table 5:** Annotation summary of the Unigenes with the seven databases.

Values	Total	Nr	Nt	SwissProt	KEGG	KOG	InterPro	GO	Intersection	Overall
Number	102,447	56,600	54,530	42,327	44,057	45,181	47,388	15,312	7,421	65,523
Percentage	100%	55.25%	53.23%	41.32%	43.00%	44.10%	46.26%	14.95%	7.24%	63.96%

### 2.5 Unigene expression

**Fig. 3:**
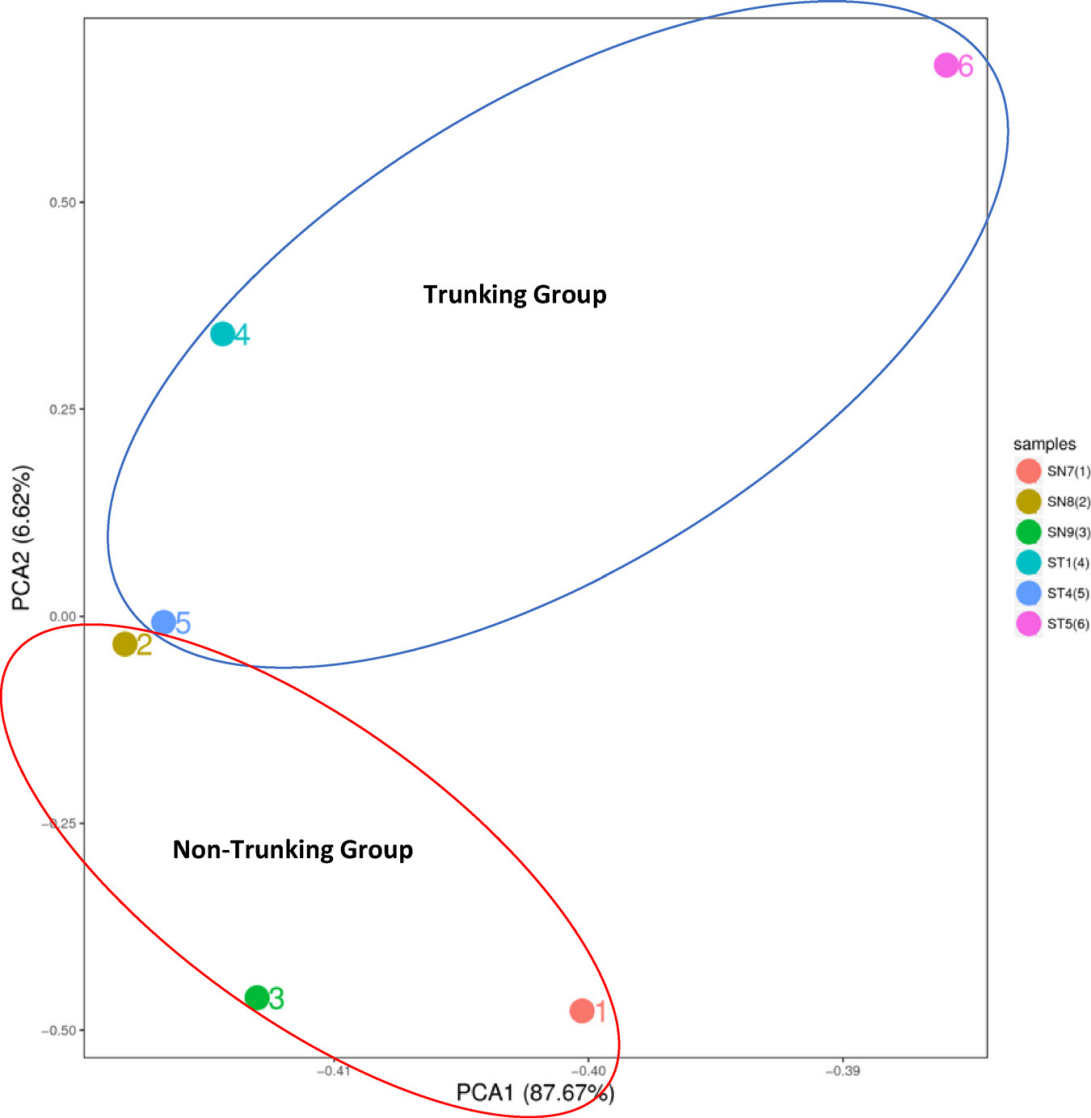
Principal component analysis of the samples gene expressions.

Based on the assembly result, the clean reads of each sample were mapped to the Unigenes with Bowtie2 software and the gene expression level were calculated with RSEM. Correlation between samples are distinguished in Principal component analysis (PCA) (Fig. 3).

Transcriptomic data of two sago phenotypes were completed, with 40.11 Gb bases sequenced, producing annotated Unigenes, and the detection of SSR and transcription factors. The data obtained from this study can be used to understand gene expression contributing to the trunking phenomenon in *M. sagu*.

## Ethics Statement

This work does not contain any studies with humans. The original collections of sago palm leaf (*M. sagu*) were made with the direct permission of Dalat Sago Plantation owned by Land Custody and Development Authority (LCDA) Holdings Sdn. Bhd., in the Mukah division. The sago palm leaf samples were not collected from any National Parks or protected wilderness areas. Additionally, the sago palm (*M. sagu*) are not endangered species.

## CRediT authorship contribution statement

**Wei-Jie Yan:** Conceptualization, Methodology, Data curation, Writing – original draft, Visualization. **Hasnain Hussain:** Conceptualization, Funding acquisition, Supervision, Writing – review & editing. **Hung Hui Chung:** Writing – review & editing. **Norzainizul Julaihi:** Funding acquisition, Writing – review & editing. **Rina Tommy:** Funding acquisition, Writing – review & editing.

## Declaration of Competing Interest

The authors declare that they have no known competing financial interests or personal relationships that could have appeared to influence the work reported in this paper.
